# Serum 25-Hydroxyvitamin D Deficiency Is Independently Associated with Cognitive Impairment, Depressive Symptoms, and Functional Dependency in Hospitalised Older Adults: A Cross-Sectional Study from Central Romania

**DOI:** 10.3390/nu18132066

**Published:** 2026-06-24

**Authors:** Valer Donca, Lucretia Avram, Tudor Cosma, Daniela Rus, Andrada Nemes, Andrei Balan, Adela Serban, Rodica Ungur, Dana Crisan

**Affiliations:** 1 Geriatrics-Gerontology, Department 5—Medical Specicalties, Faculty of Medicine, “Iuliu Hatieganu” University of Medicine and Pharmacy, 400012 Cluj-Napoca, Romania; valerdonca@gmail.com; 2 Regional Institute of Gastroenterology and Hepatology “Prof. Dr. Octavian Fodor”, Faculty of Medicine, “Iuliu Hatieganu” University of Medicine and Pharmacy, 400012 Cluj-Napoca, Romania; 3 Municipal Clinical Hospital Cluj-Napoca, Faculty of Medicine, “Iuliu Hatieganu” University of Medicine and Pharmacy, 400012 Cluj-Napoca, Romania; dr.danielarus@gmail.com; 4 Intensive Care Unit, Department 6-Surgery, Faculty of Medicine, “Iuliu Hatieganu” University of Medicine and Pharmacy, 400012 Cluj-Napoca, Romania; andrada.nemes@ymail.com (A.N.); balanandreimihai@yahoo.com (A.B.); 5 Heart Institute “Niculae Stancioiu”, Department 5—Internal Medicine, Faculty of Medicine, “Iuliu Hatieganu” University of Medicine and Pharmacy, 400012 Cluj-Napoca, Romania; adelamserban@yahoo.com; 6 Medical Rehabilitation, Department 5—Medical Specicalties, Faculty of Medicine, “Iuliu Hatieganu” University of Medicine and Pharmacy, 400012 Cluj-Napoca, Romania; rodica.ana.ungur@gmail.com; 7 Internal Medicine, Department 4-5th Medical Clinic, Faculty of Medicine, “Iuliu Hatieganu” University of Medicine and Pharmacy, 400012 Cluj-Napoca, Romania; crisan.dc@gmail.com

**Keywords:** vitamin D, elderly, cognitive impairment, late-life depression

## Abstract

**Background**: Vitamin D deficiency is highly prevalent in older adults and has been increasingly recognised as a potential contributor to cognitive decline, depressive symptomatology, and functional impairment. However, the clinical significance of specific 25-hydroxyvitamin D thresholds in relation to this multidomain geriatric phenotype remains incompletely characterised. **Methods**: We conducted a cross-sectional study of 1438 consecutive patients aged 65 years or older admitted for comprehensive geriatric assessment at a tertiary centre in Cluj-Napoca, Romania, between January 2023 and November 2025. Serum 25-hydroxyvitamin D [25(OH)D] was categorised as deficient (<20 ng/mL), insufficient (20–30 ng/mL), or sufficient (≥30 ng/mL). Cognitive function was assessed using the Montreal Cognitive Assessment (MoCA) and Mini-Mental State Examination (MMSE), depressive symptoms using the Geriatric Depression Scale (GDS-30 and GDS-SF), and functional status using Activities of Daily Living (ADL) and Instrumental Activities of Daily Living (IADL). Multivariable linear regression analyses were adjusted for age, body mass index, serum albumin, and estimated glomerular filtration rate (eGFR). **Results**: Suboptimal vitamin D status was highly prevalent in this geriatric cohort, with 43.3% of participants meeting criteria for frank deficiency (<20 ng/mL). Lower 25(OH)D concentrations were significantly associated with worse cognitive performance, greater depressive symptom burden, and higher functional dependency. Serum 25(OH)D correlated positively with MoCA and MMSE scores and inversely with ADL, IADL, and GDS scores. In adjusted models, vitamin D remained independently associated with MoCA, IADL, and GDS. Stratified analyses suggested that the main clinical deterioration occurred below 20 ng/mL, while the 20–30 ng/mL range behaved as an intermediate phenotype closer to sufficiency than to frank deficiency. **Conclusions**: In this large cohort of hospitalised older adults, serum 25(OH)D deficiency below 20 ng/mL was independently associated with poorer cognition, more depressive symptoms, and greater functional impairment. These findings support routine vitamin D assessment in geriatric practice and suggest that the <20 ng/mL threshold identifies a clinically relevant high-risk phenotype.

## 1. Introduction

The global ageing of the population is bringing neurocognitive disorders and late-life depression to the fore of clinical medicine and public health [1]. Dementia prevalence is projected to exceed 150 million by 2050, while depression already affects up to 20% of community-dwelling and up to 40% of institutionalised older adults, and remains substantially underdiagnosed in this population [2,3]. Among modifiable biological contributors, vitamin D has attracted increasing attention. Its nuclear receptors, vitamin D receptors (VDR) and the activating enzyme CYP27B1 are widely expressed in the hippocampus, prefrontal cortex, and amygdala, enabling local synthesis of 1,25(OH)_2_D_3_ and direct regulation of neuronal survival, neuroplasticity, and neuropsychiatric function [4,5].

Hypovitaminosis D is highly prevalent in the geriatric population, driven primarily by age-related declines in cutaneous synthesis, limited sun exposure, and dietary inadequacies [4]. In Romania, national surveillance data indicate a deficiency prevalence of 39.83% among high-risk adults, highlighting the regional public health relevance of this issue [6].

Recent reviews suggest that vitamin D deficiency may be associated with altered brain-derived neurotrophic factor (BDNF) signalling and increased inflammatory activity, both of which have been linked to cognitive and mood decline in older adults [7]. Randomised controlled trials and systematic meta-analyses have yielded modest and heterogeneous evidence regarding the effects of vitamin D supplementation on cognitive performance, depressive symptomatology, and functional status in older adults. Notably, the most consistent benefits have been observed in individuals with baseline deficiency, suggesting that targeted correction of frank hypovitaminosis D—rather than supplementation in vitamin D-replete populations—may constitute the clinically meaningful therapeutic window [8].

Epidemiological data consistently link low 25-hydroxyvitamin D [25(OH)D] levels to poorer executive function, accelerated cognitive decline, and elevated depressive symptoms [9,10,11]. Crucially, late-life depression and cognitive impairment often coexist and mutually reinforce one another through shared neurodegenerative and vascular pathways, forming a self-amplifying pathological triad in which vitamin D represents a potentially modifiable factor [12,13]. Furthermore, serum vitamin B12 was included as a secondary continuous covariate in the regression analyses, given its overlapping metabolic pathways in homocysteine homeostasis and neuronal methylation, both of which independently contribute to cognitive frailty in older adults. This inclusion is supported by large-scale population data indicating that the neurocognitive risk associated with hypovitaminosis D is further amplified in the presence of co-occurring nutritional deficits [14]. Despite these well-established pathways, data evaluating the concurrent cognitive and affective burden within a single, highly characterised geriatric cohort remain scarce, with Central and Eastern European populations significantly underrepresented in the international literature [6].

To address these gaps, this cross-sectional study examined the relationship between serum 25(OH)D status and neurocognitive/functional outcomes in a large cohort of 1438 patients aged ≥65 years evaluated at a tertiary geriatric centre in Cluj-Napoca, Romania. Specifically, we assessed how serum 25(OH)D concentrations correlate with performance on the Montreal Cognitive Assessment (MoCA, primary outcome), the Mini-Mental State Examination (MMSE), and the Geriatric Depression Scale (GDS-15). To prevent confounding, we controlled for global nutritional indices, including body mass index (BMI), functional independence, and anaemia parameters, while incorporating serum vitamin B12 levels strictly as a secondary continuous covariate in our multivariate regression models rather than a primary exposure pathway.

## 2. Materials and Methods

### 2.1. Study Design and Population

This cross-sectional observational study was conducted at the Clinical Geriatrics Department of Cluj-Napoca Municipal Clinical Hospital, Romania, between January 2023 and November 2025. A total of 1438 consecutive patients aged ≥65 years admitted for comprehensive geriatric assessment (CGA) were enrolled. Demographic data including age, sex, educational attainment, place of residence, and marital status were systematically collected for each participant. All assessments were completed within the same hospitalisation episode, typically within 48 to 72 h of admission, following a standardised sequence: collection of clinical history and demographic data, venous blood sampling, functional evaluation, cognitive screening, and affective assessment. Patients were included if they were able to complete the full assessment protocol; exclusions comprised acute illness, delirium, terminal illness, severe dementia, active psychosis, and major uncorrected sensory impairment. The study was conducted in full accordance with the Declaration of Helsinki and approved by the Institutional Review Board of the Municipal Clinical Hospital Cluj-Napoca (Protocol No. 29/2020; date of approval: 16 October 2020). Written informed consent was obtained from all participants prior to enrolment.

### 2.2. Laboratory Evaluation

All participants underwent a standardised haematologic and biochemical panel tailored to the geriatric population, comprising: complete blood count (CBC); an anaemia characterisation panel (serum iron, ferritin, transferrin, transferrin saturation); folic acid; serum vitamin B12 (reference range 187–883 pg/mL), included as a secondary continuous variable given its overlapping metabolic pathways with vitamin D and its independent association with cognitive frailty [14]; inflammatory markers (CRP, fibrinogen); renal function (creatinine, eGFR by the European Kidney Function Consortium [EKFC] equation, cystatin C, urea); hepatic function (ALT, AST, GGT, bilirubin, albumin); electrolytes and thyroid function (TSH and free T4). Serum 25-hydroxyvitamin D was measured by chemiluminescence immunoassay on a fully automated Cobas e801 analyzer (Roche Diagnostics GmbH, Mannheim, Germany) at the central laboratory of the Municipal Clinical Hospital Cluj-Napoca, where all samples were processed under standardised conditions. Measurements were performed on samples collected during the same hospitalisation episode as the clinical assessments. Serum 25(OH)D concentrations were classified in accordance with the 2011 Endocrine Society Clinical Practice Guideline as: deficient (<20 ng/mL), insufficient (20–30 ng/mL), or sufficient (≥30 ng/mL).

### 2.3. Functional Autonomy Assessment

Functional independence was assessed by a geriatrician through structured direct interview with the patient. Collateral input from accompanying caregivers or family members was sought where necessary. Basic self-care capacity was evaluated using the Activities of Daily Living (ADL) scale, encompassing bathing, dressing, feeding, and mobility. Complex daily functioning was assessed using the Instrumental Activities of Daily Living (IADL) scale, covering tasks such as managing finances, medication use, transportation, and household activities [15,16]. For each item, performance was rated on a graduated scale reflecting the degree of assistance required, from full independence to partial, substantial, or complete dependence.

### 2.4. Cognitive Assessment

Cognitive status was evaluated using two internationally validated instruments administered during the same hospitalisation episode. The Montreal Cognitive Assessment (MoCA) was administered by a geriatrician. It is a 30-point screening instrument with superior sensitivity for mild cognitive impairment, encompassing executive function, visuospatial abilities, attention, language, abstraction, memory, and orientation [17]. One point was added for patients with ≤12 years of formal education. Cognitive categories were defined as: normal (26–30), mild impairment (18–25), moderate impairment (10–17), and severe impairment (0–9). MoCA was designated as the primary cognitive outcome given its superior sensitivity to early executive and attentional deficits—the domains most consistently linked to vitamin D insufficiency in longitudinal data. The Mini-Mental State Examination [18] was administered by a trained clinical psychologist, the same assessor across all participants, serving as the secondary cognitive outcome (19). It assesses orientation, registration, attention, recall, and language on a 30-point scale. Patients were stratified as: normal (24–30), mild impairment (21–23), moderate impairment (11–20), and severe impairment (0–10).

### 2.5. Affective Assessment

Depressive symptomatology was assessed by a geriatrician using GDS, an instrument developed and validated specifically for the older adult population [19].

Both validated forms were administered to all eligible participants during the same hospitalisation episode. The GDS Long Form (GDS-30) is a 30-item yes/no questionnaire with documented sensitivity of 92% and specificity of 89%, scored as: normal (0–9), mild/moderate depression (10–19), and severe depression (20–30). The abbreviated GDS Short Form (GDS-SF, 15 items) [20] was scored as: normal (0–4), mild depression (5–8), moderate depression (9–11), and severe depression (12–15). The concurrent administration of both forms strengthened the reliability of affective categorisation and permitted cross-validation between instruments.

### 2.6. Patient Subgrouping

Participants were stratified into three groups according to serum 25(OH)D concentration: deficient (<20 ng/mL, *n* = 623), insufficient (20–30 ng/mL, *n* = 361), and sufficient (>30 ng/mL, *n* = 454). To assess the clinical impact across the full range of vitamin D status and to identify which segment of the suboptimal range drives the principal deterioration in cognitive, affective, and functional parameters, four pre-specified pairwise comparisons were performed: (1) non-sufficient vs. sufficient (<30 vs. ≥30 ng/mL); (2) deficient vs. non-deficient (<20 vs. ≥20 ng/mL); (3) insufficient vs. sufficient (20–30 vs. >30 ng/mL); and (4) deficient vs. insufficient (<20 vs. 20–30 ng/mL).

### 2.7. Statistical Analysis

Statistical analyses were performed using IBM SPSS Statistics, version 31.1 (IBM Corp., Armonk, NY, USA). Continuous variables were not normally distributed on Shapiro–Wilk testing and are therefore reported as median and interquartile range (IQR), and compared between groups using the Mann–Whitney U test; categorical variables are reported as frequencies and percentages and compared using the Chi Square test χ^2^ test. Spearman rank correlation coefficients were used to assess associations between serum 25(OH)D and continuous clinical outcomes. Three multivariate linear regression models were fitted for the principal cognitive (MoCA), functional (IADL) and affective (GDS-30) outcomes, with serum 25(OH)D entered as a continuous predictor and each model adjusted for a pre-defined set of covariates known to be related to both vitamin D metabolism and the outcomes of interest: age, BMI, serum albumin and eGFR-EKFC. Model assumptions were checked, and multicollinearity was assessed using variance inflation factors (VIF), all of which remained below 2. Model assumptions were checked. Missing data were handled by complete-case analysis. All *p*-values are two-sided, with statistical significance set at *p* < 0.05.

## 3. Results

### 3.1. Baseline Characteristics of the Study Population

The final analytical cohort comprised 1438 elderly patients aged 65 years or older (median age 80.5 years [IQR 74.7–85.5]; 71.1% female). Applying clinical consensus thresholds, 43.3% (*n* = 623) of the patients were classified as vitamin D deficient (<20 ng/mL), 25.1% (*n* = 361) as insufficient (20–30 ng/mL), and 31.6% (*n* = 454) as sufficient (≥30 ng/mL). The median serum 25(OH)D for the overall cohort was 22.59 ng/mL [IQR 13.21–33.09], aligning with previously reported national surveillance data for high-risk Romanian adults. The baseline demographic, biochemical, psychometric, and functional characteristics of the total population, stratified according to serum 25-hydroxyvitamin D status, are systematically summarised in Table 1. Only a minor fraction of the evaluated patients exhibited fully sufficient serum 25(OH)D concentrations, underscoring hypovitaminosis D as a pervasive clinical challenge in this hospitalised geriatric population.

### 3.2. Clinical and Biochemical Characteristics According to Vitamin D Status

Stratification at the 30 ng/mL sufficiency threshold revealed consistent multi-domain differences between non-sufficient and sufficient participants (Table 2). Non-sufficient patients showed significantly greater functional dependency on ADL and IADL scales, higher depressive burden on both GDS forms, lower MoCA and MMSE scores, and a compromised biochemical profile—lower haemoglobin, albumin, and vitamin B12, alongside elevated CRP and cystatin C concentrations. These differences were further amplified when the cohort was dichotomized at the strict deficiency threshold of 20 ng/mL (Table 3). Deficient patients were significantly older and showed uniformly worse functional, cognitive, and affective outcomes, clustered with more pronounced anaemia markers, visceral protein depletion, reduced eGFR, and heightened systemic inflammation. Direct comparison between the insufficient and sufficient subgroups showed that these two populations were clinically and biochemically comparable across the major outcome domains (Table 4): no significant differences were found for ADL, IADL, MoCA, MMSE, or GDS-30, with only BMI and GDS-SF reaching significance. By contrast, direct comparison between the deficient and insufficient subgroups revealed substantial clinical gradients across functional and cognitive parameters (Table 5), while absolute depressive symptom scores did not differ significantly between these two sub-cohorts.

### 3.3. Correlations Between Serum 25(OH)D and Clinical Outcomes

Bivariate Spearman rank correlation analysis was performed to explore the continuous relationships between serum 25(OH)D concentrations and psychometric/functional outputs (Table 6). Higher 25(OH)D levels were consistently associated with better cognitive and functional outcomes and lower depressive symptom scores, although the correlation coefficients were modest in magnitude. According to Cohen’s standard benchmarks for behavioural and clinical sciences, serum 25(OH)D concentrations exhibited a small-to-moderate positive association with cognitive performance as measured by the MoCA instrument (r_s_ = 0.232, *p* < 0.001). In contrast, the associations with the MMSE score (r_s_ = 0.149, *p* < 0.001) and functional independence via the IADL score (r_s_ = 0.134, *p* < 0.001) were weak, albeit highly statistically significant due to the large sample size. Similarly, a weak but statistically significant inverse correlation was observed between serum 25(OH)D and affective burden on the GDS-15 scale (r_s_ = −0.098, *p* < 0.001).

### 3.4. Independent Associations of Vitamin D in Multivariate Models

Multivariate linear regression adjusting for age, BMI, serum albumin, and eGFR-EKFC showed that serum 25(OH)D remained an independent predictor across all three primary outcomes. In terms of standardised effect size, the association was strongest for cognitive performance and functional autonomy, both retaining high statistical significance (Table 7 and Table 8; both *p* < 0.001) and attenuated but still significant for depressive symptomatology (Table 9; *p* = 0.034), a pattern consistent with a graded influence of vitamin D across the cognitive–functional–affective triad. Age and serum albumin were significant co-predictors in all three models; BMI reached significance in none. These findings indicate that the association between serum 25(OH)D status and the neurocognitive, functional, and affective burden of ageing is independent of nutritional and renal status.

## 4. Discussion

In a large cohort of 1438 hospitalised geriatric patients from a Central European tertiary centre, serum 25(OH)D deficiency below 20 ng/mL was independently associated with lower cognitive performance on MoCA and MMSE, a higher depressive symptom burden on both GDS forms, and greater functional dependency on ADL and IADL scales, with all associations persisting after adjustment for age, BMI, serum albumin, and renal function. The prevalence of suboptimal vitamin D status was exceptionally high—43.3% of participants met criteria for frank deficiency (<20 ng/mL)—a pattern consistent with national surveillance data for high-risk Romanian adults [6]. The 20 ng/mL threshold established by the Endocrine Society as the boundary of clinical deficiency emerged in our data as an observed inflexion point below which multi-domain deterioration of the cognitive–affective–functional triad becomes clinically and statistically distinct.

The principal findings of this study indicate that 25(OH)D deficiency below 20 ng/mL is independently associated with lower cognitive performance, greater depressive symptom burden, and higher functional dependency, with all associations persisting after adjustment for age, nutritional status, and renal function, and that the 20 ng/mL threshold marks a clinically meaningful inflexion point below which multi-domain deterioration becomes markedly and consistently distinct.

Global cognitive performance correlated significantly and positively with serum 25(OH)D in our cohort, with the association being stronger for MoCA than for MMSE—a pattern that reflects the superior sensitivity of MoCA to executive dysfunction and attentional impairment, the cognitive domains most consistently linked to hypovitaminosis D in the recent literature [21,22]. This observation aligns with the body of epidemiological evidence accumulated over recent years, demonstrating that older patients with severe 25(OH)D deficiency score significantly lower on cognitive screening instruments and carry a substantially elevated risk of meeting criteria for cognitive impairment compared to those with adequate levels [9,23]. The neurobiological substrate of this relationship is well delineated: vitamin D receptors and the activating enzyme CYP27B1 are abundantly expressed in the hippocampus and prefrontal cortex, where active 1,25(OH)_2_D_3_ governs neuronal survival, BDNF expression, and synaptic plasticity, while deficiency suppresses these neuroprotective pathways and triggers NF-κB-dependent neuroinflammatory cascades that impair synaptic transmission and accelerate dendritic loss [24,25,26]. The retention of independent predictive value for MoCA after adjustment for age, nutritional status, and renal function supports a possible independent role of vitamin D in cognitive ageing, though the cross-sectional design cannot exclude residual confounding or reverse causality. It is also important to note that cross-sectional associations do not necessarily translate into supplementation benefit: randomised trials examining vitamin D supplementation and cognitive outcomes have produced heterogeneous results, with large trials—including the VITAL cognitive ancillary study (2000 IU/day, *n* > 3400, 2-year follow-up) and the VitaMIND RCT (24-month, *n* = 620)—reporting no significant effect on cognitive trajectories compared to placebo [27]. These findings underscore the need for prospective intervention studies specifically targeting deficient geriatric populations to determine whether correction of deficiency below 20 ng/mL modifies cognitive outcomes.

Depressive symptomatology, assessed through both forms of the GDS, showed significant negative correlations with serum 25(OH)D, and the association remained independently significant in the multivariate model, albeit of smaller magnitude than that observed for cognition and functional performance. This relative attenuation is clinically unsurprising: late-life depression is inherently multidetermined, arising from the convergence of neurobiological vulnerability with loss of autonomy, social isolation, and the burden of chronic comorbidities, dimensions that extend well beyond the influence of any single nutritional factor [3]. The direction and consistency of the association are nonetheless in keeping with a well-established epidemiological consensus, supported by meta-analyses encompassing tens of thousands of participants and by recent systematic reviews confirming the relationship between vitamin D deficiency and depressive risk across diverse adult populations and clinical settings [10,28,29,30,31,32]. Mechanistically, vitamin D sustains serotoninergic tone through upregulation of TPH2 and inhibition of SERT and MAO-A, while deficiency maintains elevated concentrations of pro-inflammatory cytokines -TNF-α, IL-6, IL-1β, that impair monoamine synthesis and promote glucocorticoid resistance, a recognised neurochemical substrate of treatment-resistant late-life depression [33,34]. That age and serum albumin independently co-determined depressive scores in the multivariate model further underscores the synergistic nature of biological ageing and nutritional depletion: vitamin D may be associated with geriatric affective risk as part of a broader multi-nutritional vulnerability profile rather than as an isolated determinant. Notably, depressive scores did not differ significantly between the deficient and insufficient subgroups, unlike cognitive and functional parameters, suggesting that the affective dimension follows a more continuous gradient in relation to 25(OH)D levels and is less sharply sensitive to the 20 ng/mL threshold.

The pre-specified stratified analysis across four pairwise comparisons allowed precise localisation of the clinical inflexion point. Participants in the insufficiency range (20–30 ng/mL) and those with sufficient levels (≥30 ng/mL) proved clinically and biochemically comparable across cognitive function, functional autonomy, and major depressive symptoms, whereas the deficient subgroup (<20 ng/mL) diverged systematically, showing significant deterioration across all these parameters relative to the insufficient subgroup. The 20 ng/mL threshold established by Endocrine Society guidelines as the boundary of clinical deficiency [35] thus emerges, in our cohort, as a genuine point of fracture: below this value, nuclear VDR occupancy falls beneath the level required for optimal transcriptional regulation of neuroprotective gene networks, calcium homeostasis fails, and secondary hyperparathyroidism ensues, with direct cerebral consequences including reduced neurotrophic support, accelerated amyloid-β accumulation, and compromised cerebrovascular endothelial integrity [9,36,37].

The strongest statistical signal in the dataset was observed for functional autonomy. Both ADL and IADL scales yielded the largest effect sizes, with associations persisting robustly in the fully adjusted multivariate models. The predominance of the vitamin D effect on IADL over ADL is consistent with the dual cognitive and physical demands inherent to instrumental tasks, which require simultaneously intact prefrontal function and adequate neuromuscular capacity—both domains known to be vulnerable to hypovitaminosis D [38].

Age emerged, as anticipated, as the dominant independent predictor across all primary outcomes, reinforcing the notion that vitamin D operates as a modifiable risk factor superimposed upon the inevitable biological substrate of ageing. Serum albumin contributed independently and significantly to all three regression models, functioning as a positive determinant of cognitive performance and a protective factor against both functional dependency and depressive burden [39]. Collectively, these findings suggest that nutritional depletion acts as a potent amplifier of neurocognitive vulnerability in the hospitalised geriatric population.

The lower vitamin B12 concentrations observed in the deficient subgroup point to a broader clustering of micronutritional deficits sharing overlapping metabolic pathways. Both homocysteine homeostasis and neuronal methylation are implicated in this interplay, and their combined neurocognitive impact is likely to exceed the sum of individual contributions [14,40].

At the level of biochemical biomarkers, higher CRP concentrations and lower haemoglobin and albumin levels in the deficient subgroup support a model in which hypovitaminosis D acts both as a driver and amplifier of systemic inflammation, malnutrition, and anaemia. The well-documented immunomodulatory role of 1,25(OH)_2_D_3_, through suppression of NF-κB transcriptional activity and downregulation of pro-inflammatory cytokine production, provides a direct mechanistic foundation for these observations [7,26], while low-grade chronic inflammation in turn contributes to cognitive decline through microglial activation and synaptic injury [2]. Coexisting anaemia introduces a complementary mechanism, reduced oxygen delivery to the metabolically intensive cortical and hippocampal regions, whose clinical relevance in the cascade of cognitive deterioration in the frail older patient has been documented independently of vitamin D status [12,13,41].

The principal strengths of this study include the substantial sample size, the concurrent use of four validated instruments two cognitive (MoCA, MMSE) and two affective (GDS-30, GDS-SF) the pre-specified four-way threshold stratification strategy that enables precise localisation of the clinical inflexion point, and the Central and Eastern European geographic setting, which remains systematically underrepresented in the international literature. Several limitations must be acknowledged explicitly. First, the cross-sectional design precludes causal inference and cannot exclude reverse causality-cognitive decline or depression-associated lifestyle changes, including reduced sun exposure and dietary diversity, may independently drive lower vitamin D status. Second, the absence of data on supplementation history, dietary vitamin D intake, and seasonality of blood sampling represents a further constraint. Third, this study was conducted in a hospitalised geriatric population undergoing comprehensive assessment, which is a clinically specific and relatively unwell group; generalisability to community-dwelling older adults is therefore limited. Fourth, the exclusion criteria systematically removed patients with severe dementia and delirium—the most cognitively impaired individuals—which likely attenuates the observed associations. Fifth, four pairwise comparisons were performed across multiple outcomes without correction for multiple comparisons; no Bonferroni or false discovery rate adjustment was applied, and the family-wise error rate should be considered when interpreting subgroup-level *p*-values. Finally, educational attainment could not be included as a continuous covariate in the regression models, though the MoCA scoring correction partially addresses this.

## 5. Conclusions

In this large cohort of hospitalised older adults from a Central European tertiary centre, serum 25(OH)D deficiency below 20 ng/mL was independently associated with worse cognitive, affective, and functional outcomes. These findings support the clinical relevance of vitamin D assessment in geriatric practice as an integral component of comprehensive assessments. However, given the observational nature of these data, these findings justify the implementation of prospective studies or further targeted screening to evaluate whether the targeted correction of this deficiency improves clinical outcomes in vulnerable elderly populations.

## Figures and Tables

**Table 1:** Baseline characteristics of the study population.

Parameter	Overall Cohort (*n* = 1438)	
	Median	IQR
Age (years)	80.5	[74.72–85.54]
BMI (kg/m^2^)	28.25	[23.97–32.50]
ADL	3.00	[0.00–14.00]
IADL	11.00	[3.00–18.00]
GDS	12.00	[7.00–18.00]
MoCA	21.00	[16.00–26.00]
MMSE	24.00	[20.00–28.00]
Haemoglobin (g/dL)	12.90	[11.50–14.00]
Serum albumin (g/dL)	4.20	[3.90–4.50]
eGFR EKFC (mL/min/1.73 m^2^)	73.00	[58.70–80.13]
Cystatin C (mg/L)	1.40	[1.20–1.80]
Vitamin D (ng/mL)	22.59	[13.21–33.09]
Vitamin B12 (pg/mL)	343.15	[256.81–486.00]
C–reactive protein (mg/L)	0.44	[0.16–1.49]
**Sex**	***n***	**%**
Female	1022	71.1
Male	416	28.9
**Marital status**	***n***	**%**
Single	28	1.9
Married	469	32.6
Divorced	52	3.6
Widowed	881	61.6
**Smoking**	***n***	**%**
Never smoker	1108	77
Former smoker	250	17.5
Current smoker	80	5.6
**Alcohol consumption**	***n***	**%**
No alcohol consumption	1145	79.6
>1 time per month	293	20.4
**Residence**	***n***	**%**
Rural	568	39.5
Urban	870	60.5

Values are presented as the median and interquartile range (IQR) or percentage (%). Abbreviations: IQR—Interquartile Range, BMI—Body Mass Index, ADL—Activities of Daily Living, IADL—Instrumental Activities of Daily Living, GDS—Geriatric Depression Scale, MoCA—Montreal Cognitive Assessment, MMSE—Mini Mental State Examination, eGFR—estimated Glomerular Filtration Rate, EKFC—European Kidney Function Consortium.

**Table 2:** Comparison of demographic, clinical, and biochemical characteristics according to vitamin D status (<30 vs. ≥30 ng/mL).

Parameter	Vitamin D < 30 ng/mL (*n* = 984)		Vitamin D ≥ 30 ng/mL (*n* = 454)		*p*-Value
	Median	IQR	Median	IQR	
Age (years)	80.58	[75.06–85.55]	80.09	[74.23–85.62]	0.303
BMI (kg/m^2^)	28.61	[24.49–32.63]	27.71	[23.33–32.12]	0.016
ADL	4	[0.00–16.50]	2	[0.00–9.00]	<0.001
IADL	13	[4.00–20.00]	9	[1.00–16.00]	<0.001
GDS	13	[7.00–18.00]	11	[6.00–17.00]	0.003
GDS SF	6	[3.00–10.00]	5	[3.00–9.00]	<0.001
MoCA	20	[15.00–25.00]	23	[18.00–27.00]	<0.001
MMSE	24	[20.00–28.00]	26	[21.00–28.00]	0.048
Haemoglobin (g/dL)	12.90	[11.40–14.00]	13.00	[11.90–14.00]	0.035
Serum albumin (g/dL)	4.20	[3.80–4.50]	4.30	[4.00–4.60]	<0.001
eGFR EKFC (mL/min/1.73 m^2^)	72.18	[57.74–80.11]	73.69	[60.78–80.21]	0.572
Cystatin C (mg/L)	1.50	[1.20–1.90]	1.40	[1.10–1.70]	0.046
Vitamin B12 (pg/mL)	331.84	[246.99–475.50]	364.85	[271.87–517.36]	0.001
C–reactive protein (mg/L)	0.48	[0.17–1.53]	0.34	[0.14–1.04]	0.016

Continuous variables are presented as median (IQR) and compared using the Mann–Whitney U test. Abbreviations: IQR—Interquartile Range, BMI—Body Mass Index, ADL—Activities of Daily Living, IADL—Instrumental Activities of Daily Living, GDS—Geriatric Depression Scale, GDS SF—Geriatric Depression Scale Short Form, MoCA—Montreal Cognitive Assessment, MMSE—Mini Mental State Examination, eGFR—estimated Glomerular Filtration Rate, EKFC—European Kidney Function Consortium.

**Table 3:** Comparison of demographic, clinical, and biochemical characteristics according to vitamin D status (<20 vs. ≥20 ng/mL).

Parameter	Vitamin D < 20 ng/mL (*n* = 623)		Vitamin D ≥ 20 ng/mL (*n* = 815)		*p*-Value
	Median	IQR	Median	IQR	
Age (years)	81.80	[76.44–86.58]	79.53	[73.81–85.22]	<0.001
BMI (kg/m^2^)	28.32	[23.87–32.63]	28.25	[24.16–32.36]	0.933
ADL	6	[0.75–22.00]	2	[0.00–10.00]	<0.001
IADL	15	[7.00–20.00]	9	[1.00–17.00]	<0.001
GDS	13	[7.00–18.00]	11	[7.00–17.00]	0.017
GDS SF	7	[4.00–10.00]	5	[3.00–9.00]	<0.001
MoCA	19	[14.00–24.00]	22	[17.00–27.00]	<0.001
MMSE	25	[19.00–27.00]	25	[21.00–28.00]	0.005
Haemoglobin (g/dL)	12.60	[11.10–13.80]	13.10	[11.90–14.10]	<0.001
Serum albumin (g/dL)	4.10	[3.80–4.40]	4.30	[4.00–4.60]	<0.001
eGFR EKFC (mL/min/1.73 m^2^)	71.21	[56.06–78.97]	73.70	[61.47–81.16]	0.009
Cystatin C (mg/L)	1.50	[1.20–2.00]	1.40	[1.10–1.70]	0.007
Vitamin B12 (pg/mL)	320.47	[238.72–459.61]	360.39	[266.71–513.23]	<0.001
C–reactive protein (mg/L)	0.51	[0.18–2.11]	0.35	[0.15–0.97]	<0.001

Continuous variables are presented as median (IQR) and compared using the Mann–Whitney U test. Abbreviations: IQR—Interquartile Range, BMI—Body Mass Index, ADL—Activities of Daily Living, IADL—Instrumental Activities of Daily Living, GDS—Geriatric Depression Scale, GDS SF—Geriatric Depression Scale Short Form, MoCA—Montreal Cognitive Assessment, MMSE—Mini Mental State Examination, eGFR—estimated Glomerular Filtration Rate, EKFC—European Kidney Function Consortium.

**Table 4:** Comparison of demographic, clinical, and biochemical characteristics according to vitamin D status (20–30 vs. ≥30 ng/mL).

Parameter	Vitamin D 20–30 ng/mL (*n* = 361)		Vitamin D ≥ 30 ng/mL (*n* = 454)		*p*-Value
	Median	IQR	Median	IQR	
Age (years)	78.5	[73.56–84.22]	80.09	[74.23–85.62]	0.040
BMI (kg/m^2^)	28.85	[25.25–32.65]	27.71	[23.33–32.12]	0.003
ADL	2	[0–10]	2	[0–9]	0.481
IADL	9	[2–17]	9	[1–16]	0.252
GDS	12	[7–18]	11	[6–17]	0.058
GDS SF	6	[3–9]	5	[3–9]	0.040
MoCA	22	[17–26]	23	[18–27]	0.572
MMSE	24	[20–28]	26	[21–28]	0.679
Haemoglobin (g/dL)	13.2	[11.90–14.15]	13	[11.90–14.00]	0.329
Serum albumin (g/dL)	4.3	[4.00–4.50]	4.3	[4.00–4.60]	0.164
eGFR EKFC (mL/min/1.73 m^2^)	73.90	[61.92–82.07]	73.69	[60.57–80.22]	0.232
Cystatin C (mg/L)	1.40	[1.20–1.80]	1.40	[1.10–1.70]	0.663
Vitamin B12 (pg/mL)	357.88	[262.13–501.88]	364.85	[271.67–517.74]	0.393
C–reactive protein (mg/L)	0.40	[0.16–0.86]	0.34	[0.14–1.04]	0.967

Continuous variables are presented as median (IQR) and compared using the Mann–Whitney U test. Abbreviations: IQR—Interquartile Range, BMI—Body Mass Index, ADL—Activities of Daily Living, IADL—Instrumental Activities of Daily Living, GDS—Geriatric Depression Scale, GDS SF—Geriatric Depression Scale Short Form, MoCA—Montreal Cognitive Assessment, MMSE—Mini Mental State Examination, eGFR—estimated Glomerular Filtration Rate, EKFC—European Kidney Function Consortium.

**Table 5:** Comparison of demographic, clinical, and biochemical characteristics according to vitamin D status (<20 vs. 20–30 ng/mL).

Parameter	Vitamin D < 20 ng/mL (*n* = 623)		Vitamin D 20–30 ng/mL (*n* = 361)		*p*-Value
	Median	IQR	Median	IQR	
Age (years)	81.80	[76.44–86.58]	78.50	[73.56–84.22]	<0.001
BMI (kg/m^2^)	28.32	[23.87–32.63]	28.85	[25.25–32.65]	0.103
ADL	6	[1–22]	2	[0–10]	<0.001
IADL	15	[7–20]	9	[2–17]	<0.001
GDS	13	[7–18]	12	[7–18]	0.398
GDS SF	7	[4–10]	6	[3–9]	0.120
MoCA	19	[14–24]	22	[17–26]	<0.001
MMSE	25	[19–27]	24	[20–28]	0.048
Haemoglobin (g/dL)	12.60	[11.10–13.80]	13.20	[11.90–14.15]	<0.001
Serum albumin (g/dL)	4.10	[3.80–4.40]	4.30	[4.00–4.50]	<0.001
eGFR EKFC (mL/min/1.73 m^2^)	71.21	[56.06–78.97]	73.90	[61.92–82.07]	0.005
Cystatin C (mg/L)	1.50	[1.20–2.00]	1.40	[1.20–1.80]	0.066
Vitamin B12 (pg/mL)	320.47	[238.72–459.61]	357.88	[262.13–501.88]	0.004
C–reactive protein (mg/L)	0.51	[0.18–2.11]	0.40	[0.16–0.86]	0.001

Continuous variables are presented as median (IQR) and compared using the Mann–Whitney U test. Abbreviations: IQR—Interquartile Range, BMI—Body Mass Index, ADL—Activities of Daily Living, IADL—Instrumental Activities of Daily Living, GDS—Geriatric Depression Scale, GDS SF—Geriatric Depression Scale Short Form, MoCA—Montreal Cognitive Assessment, MMSE—Mini Mental State Examination, eGFR—estimated Glomerular Filtration Rate, EKFC—European Kidney Function Consortium.

**Table 6:** Correlations between vitamin D levels and clinical parameters.

Parameter	Spearman	*p* Value
ADL	−0.245	<0.001
IADL	−0.261	<0.001
GDS	−0.098	<0.001
GDS SF	−0.123	<0.001
MoCA	0.232	<0.001
MMSE	0.149	<0.001

Spearman correlation coefficients are presented. Abbreviations: ADL—Activities of Daily Living, IADL—Instrumental Activities of Daily Living, GDS—Geriatric Depression Scale, GDS SF—Geriatric Depression Scale Short Form, MoCA—Montreal Cognitive Assessment, MMSE—Mini Mental State Examination.

**Table 7:** Multivariate linear regression analysis for factors associated with MoCA score.

Parameter	Adjusted B (95% CI)	Standardised β	*p* Value
Vitamin D	0.057 (0.035–0.079)	0.124	<0.001
Age (years)	−0.369 (−0.415–−0.323)	−0.434	<0.001
BMI (kg/m^2^)	0.035 (−0.013–0.083)	0.036	0.157
Serum albumin (g/dL)	1.934 (1.260–2.608)	0.143	<0.001
eGFR EKFC (mL/min/1.73 m^2^)	−0.003 (−0.016–0.011)	−0.010	0.702

Abbreviations: MoCA—Montreal Cognitive Assessment, BMI—Body Mass Index, eGFR—estimated Glomerular Filtration Rate, EKFC—European Kidney Function Consortium, CI—confidence interval. Model fit: R^2^ = 0.280; adjusted R^2^ = 0.277.

**Table 8:** Multivariate linear regression analysis for factors associated with IADL score.

Parameter	Adjusted B (95% CI)	Standardised β	*p* Value
Vitamin D	−0.069 (−0.100–−0.038)	−0.128	<0.001
Age (years)	0.409 (0.342–0.476)	0.404	<0.001
BMI (kg/m^2^)	−0.004 (−0.074–0.066)	−0.003	0.917
Serum albumin (g/dL)	−4.447 (−5.429–−3.465)	−0.267	<0.001
eGFR EKFC (mL/min/1.73 m^2^)	−0.026 (−0.048–−0.003)	−0.071	0.026

Abbreviations: IADL—Instrumental Activities of Daily Living, BMI—Body Mass Index, eGFR—estimated Glomerular Filtration Rate, EKFC—European Kidney Function Consortium, CI—confidence interval. Model fit: R^2^ = 0.367; adjusted R^2^ = 0.363.

**Table 9:** Multivariate linear regression analysis for factors associated with GDS score.

Parameter	Adjusted B (95% CI)	Standardised β	*p* Value
Vitamin D	−0.030 (−0.059–−0.002)	−0.061	0.034
Age (years)	0.119 (0.062–0.176)	0.132	<0.001
BMI (kg/m^2^)	0.037 (−0.023–0.097)	0.036	0.222
Serum albumin (g/dL)	−1.245 (−2.091–−0.399)	−0.086	0.004
eGFR EKFC (mL/min/1.73 m^2^)	−0.014 (−0.031–0.003)	−0.050	0.104

Abbreviations: GDS—Geriatric Depression Scale, BMI—Body Mass Index, eGFR—estimated Glomerular Filtration Rate, EKFC—European Kidney Function Consortium, CI—confidence interval, Model fit: R^2^ = 0.045; adjusted R^2^ = 0.041.

## Data Availability

The dataset used during the current study is available from the corresponding authors without restriction.
